# 

**Published:** 1878-01

**Authors:** 


### Books and Pamphlets Received.

Modern Medical Therapeutics. By George H. Napheys, A. M., M. D. Philadelphia: D. G. Brinton. 1878.

Modern Surgical Therapeutics. By George H. Napheys, A. M., M. D. Philadelphia: D. G. Brinton. 1878.

Diagnosis in Pathological Anatomy. By Dr. Johannes Orth. New York: Hurd & Houghton. 1878. Buffalo: Peter Paul & Co.

Public Hygiene in America. Centennial Address by Henry J. Bowditch, M. D. Boston: Little, Brown & Co. 1877.

Materia Medica. By John B. Biddle, M. D. Eighth Edition. Philadelphia: Lindsay & Blakiston. 1878. Buffalo: T. H. Butler.

Guide to Therapeutics. By Robert Farquharson, M. D. Edin. American Editor, Frank Woodbury, M. D. Philadelphia: Henry C. Lea. 1877. Buffalo: T. H. Butler.

Gonorrhœa and Syphilis. By Silas Durkee, M. D. Philadelphia: Lindsay & Blakiston. 1877. Buffalo: T. H. Butler.

Action of Medicines. By Isaac Ott, A. M., M. D. Philadelphia: Lindsay & Blakiston. 1878. Buffalo: Theo. H. Butler.

Transactions of the Medical Society of the State of Pennsylvania. Annual Session, June 13th, 1877. Philadelphia. Published by the Society. Collins, Printer. 1877.

Popular Science Monthly, and Supplement.

American Clinical Lectures. Vol. III. No. V.

Pyæmia and Septicæmia. By B. A. Watson, M, D.

Clinical Lecture on Surgery. By J. H. Pooley, M. D. Reprint from Ohio Medical and Surgical Journal.

Exposition of Facts. By A. Y. P. Garnett, M. D.

Cesarean Section after Seven Day's Labor. By Edward W. Jenks, M. D. Reprint from The American Journal of Obstetrics. October, 1877. New York: Wm. Wood & Co. 1877.

Annual Report of the Surgeon-General, U. S. Army. 1877.

The Localization of Diseased Action in the Œsophagus. By Harrison Allen, M. D. Reprint from Medical Times. Philadelphia: J. B. Lippincott & Co. 1877.

The Mechanism of Joints. By H. Allen, M. D. Extract from the Transactions of the International Medical Congress.

Report of Asylum at Walnut Hill, Hartford. 1877.

The next annual meeting of the Medical Society of the State of New York, will be held at Albany, on Tuesday, January 15th, 1878, at 11 A. M.

WM. MANLIUS SMITH, *Secretary.*

Jan 9 (2 cop)

[Vol. XVII] JANUARY, 1878. [No. 6.]

### BUFFALO

### Medical and Surgical Journal.

### Medical and Surgical Journal

PUBLISHED MONTHLY,

Containing Original Articles, Reports of Medical Societies and Hospitals, Editorial, Reviews, Correspondence, Army News, etc., etc.; including the usual variety of Medical Periodical Publications. Specimen copies sent on application.

Address Buffalo Medical & Surgical Journal, Buffalo, N. Y.

TERMS OF SUBSCRIPTION, $3.0) PER YEAR, IN ADVANCE.

All communications for publication shoud be sent before the twentieth (20th) of each month, or they will of necessity lie over until the next number. No change can be made in advertisements after the twenty-fifth. Address, Buffalo Medical and Surgical Journal, 978 Main Street.

Correspondence and original articles are solicited. Publication is no evidence that the views of the author are endorsed.

